# Supplementary material for: Improved exclusive breastfeeding rates in preterm infants after a neonatal nurse training program focusing on six breastfeeding-supportive clinical practices
Source: PLoS One. 2021 Feb 3;16(2):e0245273. doi: 10.1371/journal.pone.0245273 (PMC7857627; doi:10.1371/journal.pone.0245273)
Supplement: S1 File — (PDF) [file pone.0245273.s001.pdf]

Breastfeeding support training program for neonatal nurses

The Danish intervention study on evidence based breastfeeding support in NICUs
Seven hours of training by Ragnhild Maastrup

Content:

- The study
    - Aim, expected effects, ethics, mothers who do not breastfeed
- Breastfeeding of preterm infants
    - Official recommendations
    - Benefits and challenges for infants and mothers
- The Baby-friendly Hospital Initiative for neonatal wards (Neo-BFHI) in brief, the six practices align with Neo-BFHI recommendations
- Progression in breastfeeding/ breastfeeding milestones
- Results from the Breastfeeding survey 2009-2011 and the control group 2016-2017, results of the six practices from your NICU compared to national results and to existing evidence. Each practice ends with a brainstorm of how to facilitate implementation in your NICU.
    - Rooming-in
    - Skin-to-skin contact
    - Breastmilk expression
    - Nipple shield use
    - Pacifier use
    - Test-weighing
- Factors in infants and mothers - more vulnerable groups
- Breastfeeding history, plans, and self-efficacy – what you need to know about the mother
- Breastfeeding meetings for neonatal nurses – concept and implementation
- Parent information poster and breastmilk expression diary

The full Danish training material can be found here:

https://www.rigshospitalet.dk/afdelinger-og-klinikker/julianemarie/videnscenter-for-amning-af-boern/forskning/Documents/ammeundervisning-intervention-gn-ernæringsundersøgelsen.pdf
