# Supplementary material for: Improved exclusive breastfeeding rates in preterm infants after a neonatal nurse training program focusing on six breastfeeding-supportive clinical practices
Source: PLoS One. 2021 Feb 3;16(2):e0245273. doi: 10.1371/journal.pone.0245273 (PMC7857627; doi:10.1371/journal.pone.0245273)
Supplement: S2 File — (PDF) [file pone.0245273.s002.pdf]

Breastfeeding in the neonatal ward
What could you do?

Be as much as possible with your baby.
The more you are together, the better you will learn your baby's signals day and night.
Your baby needs to be comforted by hands he/she knows.

You should have at least 400 – 600 ml breastmilk every day for your baby, even if your baby doesn't get that much milk right now.
Each day be aware that you have enough breast milk.
A pumping diary or an app could be helpful.

Let your baby sleep skin-to-skin as often and as long as possible. Both mom and dad are good for this.

You could also make a family team of close relatives to help you with skin-to-skin holding.
If you have your baby skin-to-skin most of the time, you could have small breaks, e.g. while you eat.

When you baby should breastfeed more, you should give the pacifier less.
Every time your baby needs to suckle it should be on the breast – then your baby will learn faster.

Be patient ☺
Accept that breastfeeding is not effective in the beginning.
It will come. Practice makes perfect.
Talk to the staff about different models to transits from tube-feeding to breastfeeding and choose the one that fits you best.
