# Supplementary material for: Improved exclusive breastfeeding rates in preterm infants after a neonatal nurse training program focusing on six breastfeeding-supportive clinical practices
Source: PLoS One. 2021 Feb 3;16(2):e0245273. doi: 10.1371/journal.pone.0245273 (PMC7857627; doi:10.1371/journal.pone.0245273)
Supplement: S3 File — The nutrition study of preterm infants 2016–2019. (PDF) [file pone.0245273.s003.pdf]

The nutrition study of preterm infants 2016 - 2019

Beginning of hospitalization

Thank you for participating in the study. It is important for the study that as many as possible answers the questionnaire.

The questions in the first questionnaire are about yourself, the delivery, how the first days went, and whether you have started breast milk pumping and/or are breastfeeding. It takes approximately 10 minutes to answer the questionnaire.

If you have any questions to the questionnaire or need help, you could send a mail to the project (e-mail address).

If you cannot answer the questions exactly, you should answer as well as you remember (an approximate answer).

If you have twins, this questionnaire is for twin A and you will get another link for twin B.

The first questions are about your baby's birth (delivery).

1. When was your baby born? Date: _____________
2. The gestational age of your baby at birth: ______weeks _______days
3. Birth weight: _____________ grams
4. Your baby is:

   A girl

   A boy

5. Your baby was born by Caesarean section?

   Yes

   No

6. Did you have complications in connection to labour/delivery, which prevented you, for more than the first 24 hours, from being together with your baby?

   Yes

   No

7. When did you or your partner participate in the care of your baby for the first time (eg. Comfort or calm your baby, change diaper, measure temperature)?

   Within 6 hours after delivery

   Between 6 and 24 hours after delivery

   Later than 24 hours after delivery

The next questions are about your experiences and thoughts about breastfeeding.

8. Did you plan to breastfeed your baby?

   Yes (*Proceed to question **10***)

   No

   I don't know

9. What is your reason you did not plan to breastfeed?
   *(Please answer this question and proceed to question **13**)*

   I do not want to breastfeed

   I cannot breastfeed (e.g. because of breast surgery)

   I am not allowed to breastfeed (e.g. because of medication)

   I have not decided yet

   Other reasons
   Please describe: ___________________________________

10. For how long have you planned to breastfeed your baby?

Shorter than one month

1 - 4 months

4 - 6 months

6 - 12 months

12 - 24 months

More than 24 months

11. Of how great importance is it to you to breastfeed?

Very great importance

Great importance

Some importance

Little importance

No importance

I don’t know

12. How confident are you that you can breastfeed your preterm baby for as long as you have planned?

Very confident

Confident

Don’t know

Uncertain

Very uncertain

13. Does your partner support your choice of breastfeeding?

Yes

No

I don’t know

I’m alone with my baby

14. Have you breastfed before?

Yes

No, this is my first child
(Proceed *to question* ***17***)

No, I haven’t breastfed my other children
(Proceed *to question* ***17)***

15. For how long have you breastfed your first child without giving anything else than your milk?

Shorter than one month

1 - 4 months

4 - 6 months

More than 6 months

16. For how long have you breastfed your first child in total?

Shorter than one month

1 - 4 months

4 - 6 months

6 - 12 months

12 - 24 months

More than 24 months

17. Did you have a conversation on the neonatal ward about your plans and wishes for breastfeeding/feeding and any previous breastfeeding problems?

Yes

No

18. Has your baby been put to the breast (mouth against the nipple or latched on - not necessarily sucking or sinking, we call it first breastfeeding attempt)

Yes

No (Proceed *to question **22)***

19. When was your baby's first breastfeeding attempt? Date: ___________
What was your baby's weigh this day? Grams: __________

20. What did your baby do at the first breastfeeding attempt? Please choose the best performance you baby showed at the first breastfeeding attempt.

Smells the breast

Licks and tastes the milk

Seeks and finds breast, gets nipple in mouth

Suckles and swallows briefly
Breastfeeds more effectively, reduced amounts

through feeding tube/cup
Breastfeeds larger amounts than is given through

feeding tube/cup

Breastfeeds a complete feed

Don't know

21. At the same time did your infant then get: *(Please answer all 3 questions with a tick in a box on each line)*

	Yes	No
a. Nasal CPAP?		
b. Oxygenation?		
c. Break from nasal CPAP or oxygen?		

22. Today, your baby is ________ days old.

23. Yesterday your baby was placed in:

A closed incubator

An open incubator

A cot/bed

Questions about breast milk pumping

24. Have you started breast milk pumping/expressing for your baby?

Yes

No *(Proceed to question **30**)*

25. When was your breast at first stimulated for lactation after your baby's birth (by stimulating we mean the first time you breastfed or expressed by hand, or pumped)?

Before my baby was 6 hours old

When my baby was 6 – 12 hours old

When my baby was 12 – 24 hours old

When my baby was 24 – 48 hours old

When my baby was more than 48 hours old

26. Which method did you use the first time your breast was stimulated?

Handexpression

Breastpump (could be electric or manual)

My baby was breastfed

27. How many times have you pumped for the last 24 hours? _________ times

28. Did you ever pump/express 400 ml in 24 hours?

Yes

No *(Proceed to question **30**)*

29. When did you for the first time pump/express 400 ml in 24 hours?

Date:_______________________

30. How is your baby being fed today? (*You may tick more than one box)*

Breastfeeding

Feeding tube

Cup

Lact-aid

Finger-feeding

Bottle

Intravenous nutrition

Questions about skin-to-skin contact

With skin-to-skin contact we mean that your baby is only dressed in a nappy, maybe a cap and socks, and maybe an open blouse, but in a way that your baby's stomach, chest and legs are in direct contact with your (or another adult's) bare chest.

31. Have your baby been skin-to-skin with you and/or your partner for a minimum of 60 minutes (in total) within the first six hours of birth?

Yes

No

32. When did you (the mother) at first have your baby skin-to-skin?

Immediately after the baby was born

Short time after delivery = 0 – 6 hours

6 – 24 hours after delivery

1 – 2 days after delivery

More than two days after delivery

My baby has not been skin-to-skin with me

33. When did your partner (or another adult) at first have your baby skin-to-skin?

Immediately after the baby was born

Short time after delivery = 0 – 6 hours

6 – 24 hours after delivery

1 – 2 days after delivery

More than two days after delivery

My baby has not been skin-to-skin with my partner (or another adult)

34. For how long did your baby have skin-to-skin contact yesterday?
(You are supposed to add the hours, if your baby was skin-to-skin with persons other than yourself)

0 – 1 hours

1 – 2 hours

2 – 4 hours

4 – 6 hours

6 – 8 hours

8 – 12 hours

12 - 20 hours

More than 20 hours

My baby did not have skin-to-skin contact yesterday

General questions about you

35. How old are you? ____________ years

36. How tall are you? (what is your height?) ____________ cm

37. How much did you weigh before your pregnancy? ____________ kg

38. How do you live?

Together with my baby's father

Together with an adult other than my baby's father

Alone

39. Do you have other children at home (apart from your new-born baby)?

Yes

No

If yes, please give the number (apart from your new-born baby) ___________

and the age of your youngest child (apart from your new-born baby) __________

40. In which country are you born? _________________

41. Which language do you speak at home? _________________

42. Which educational courses/programmes have you completed or are you taking?

None

Labour-market courses, special training programmes

Occupational programmes (apprenticeship, traineeship e.g. carpentry, welding)

Short secondary educational programmes (2-3 years)

Medium-length secondary educational programmes (3-4 years)

Long secondary educational programmes (4-6 years or longer)

43. Have you ever received breast surgery?

Yes, I have or have had a breast nipple piercing

Yes, my breast(s) has been diminished (something has been removed)

Yes, my breast(s) has been enlarged (implants)

No

44. Do you smoke?

Yes

No

Thank you for filling out the questionnaire.
You will receive the next questionnaire when your baby is discharged to home. You will be asked when you baby took all feeds orally and when you baby was discharged so please pay attention to these dates.

The survey is done in cooperation with
Knowledge Centre for Breastfeeding Infants with Special Needs at Copenhagen University Hospital, Rigshospitalet and neonatal wards in Denmark.
