# Supplementary material for: Improved exclusive breastfeeding rates in preterm infants after a neonatal nurse training program focusing on six breastfeeding-supportive clinical practices
Source: PLoS One. 2021 Feb 3;16(2):e0245273. doi: 10.1371/journal.pone.0245273 (PMC7857627; doi:10.1371/journal.pone.0245273)
Supplement: S4 File — The nutrition study of preterm infants 2016–2019. (PDF) [file pone.0245273.s004.pdf]

The nutrition study of preterm infants 2016 - 2019

Discharge

Thank you for participating in the study. It is important to the study that as many as possible answers the questionnaire.

This questionnaire is about how your baby has started oral feeding, and about your infant's hospital stay, as well as the breastfeeding support you have received. It takes approximately 15 minutes to answer the questionnaire.

If you have any questions to the questionnaire or need help, you could send a mail to the project (e-mail address).

If you cannot answer the questions exactly, you should answer as well as you remember (an approximate answer).

Questions about your baby's first breastfeeding experience

1. Date for answering the questionnaire: ________________

2. Has your baby been put to the breast (mouth against the nipple or latched on - not necessarily sucking or sinking, we call it first breastfeeding attempt)

   Yes

   No (Proceed *to question* ***8)***

   I did answer this question previously (Proceed *to question* ***8)***

3. When was your baby's first breastfeeding attempt? Date: ___________
   What was your baby's weigh this day? Grams __________

4. What did your baby do at the first breastfeeding attempt? Please choose the best performance you baby showed at the first breastfeeding attempt.

   Smells the breast

   Licks and taste the milk

   Seeks and finds breast, gets nipple in mouth

   Suckles and swallows briefly

   Breastfeeds more effectively, reduced amounts through feeding tube/cup

   Breastfeeds larger amounts than is given through feeding tube/cup

   Breastfeeds a complete feed

5. At the same time did your infant then get: *(Please answer all 3 questions with a tick in a box on each line)*

	Yes	No
a. Nasal CPAP?	1	2
b. Oxygenation?	1	2
c. Break from nasal CPAP or oxygen?	1	2

8. When did your baby take all feeding orally without tube-feeding? Date: _________
   What was your baby's weigh this day? Grams __________

9. How did your baby feed this day?

My baby was only breastfed	1
My baby was both breastfed and bottle-fed	2
My baby was only bottle-fed	3
Other (eg. Cup, lact-aid)	4

   Please describe: ____

10. Did your baby need tube-feeding again later on?

Yes 1

No *(Proceed to question **13**)* 2

11. If yes, since what date has your baby been completely without tube-feedings?

Since date____________

12. How did your baby feed this day?

My baby was only breastfed 1

My baby was both breastfed and bottle-fed 2

My baby was only bottle-fed 3

Other (eg. Cup, lact-aid) 4

Please describe: ____

13. Did you receive a pamphlet about breastfeeding preterm infants?

Yes 1

No *(Proceed to question **15**)* 2

14. Was the pamphlet a help?

No help 1

A little help 2

Some help 3

A great help 4

A very great help 4

Questions about how your baby is fed at the final discharge from hospital

15. When was your baby discharged from hospital? (If your baby has been tube-fed at home, please give the date for ending the "Early discharge program")
Date: ___________

16. Your baby's weight at the final discharge: _____________ grams (approx.)

17. How was your baby fed at the final discharge?

My baby was only breastfed	1
My baby was both breastfed and bottle-fed	2
My baby was only bottle-fed *(go to question **19)***	3
Other (eg. Cup, lact-aid)<br>Please describe**:** ______________________________	4

18. How confident are you that you can breastfeed your preterm baby for as long as you have planned?

Very confident	1
Confident	2
Don't know	3
Uncertain	4
Very uncertain	5

19. Did you experience any of the following breastfeeding problems? You may choose more than one answer

Too little milk (after the first week)	1
Sore nipples because of wounds/cracks (pain)	2
Mastitis	3
Inverted/flat nipples	4
Breasts too engorged for baby to latch	5
My baby was not getting satisfied by breastfeeding	6
My baby was not gaining enough weight by breastfeeding	7
My baby could not/would not breastfeed	5
My baby could not open his/her mouth high enough to latch on	4
My baby slipped the nipple	1
My baby became frustrated at the breast	2
My baby fell asleep at the breast	3
My baby could not/would not breastfeed	5

My baby could not/would not breastfeed 5

My baby could not/would not breastfeed 5

It is hard not to know exactly how much my baby gets 8

Other 10
Please describe: ____

I have not experienced any breastfeeding probelms 5

My baby has not been put to the breast/breastfed 5

For every chosen breastfeeding problem this question was repeated:
When did you for the first time experience (this problem)? Date _______
Is this still a problem?

Yes 1

No 2

My baby is not breastfed (when this is relevant for the answer) 1

You only need to answer question 20 – 23 if your baby is receiving milk in a way other than by breastfeeding.

20. What does your baby get from the bottle (cup or alike)?

Expressed breast milk 1

Infant formula 2

Both expressed breast milk and formula 3

21. What is the main reason that your baby is not fully breastfed at discharge?
(Choose only one main reason, in the next question you are given the possibility to add contributing reasons)

I did not have enough milk 1

I've stopped because of wounds, cracks or sore nipples 2

I've stopped because of mastitis 3

I have inverted/flat nipples 4

My baby could not/would not breastfeed 5

My baby was not getting satisfied by breastfeeding 6

My baby was not gaining enough weight by breastfeeding 7

I need to know exactly how much my baby gets 8

I feel bound to breastfeeding 9

Other 10
Please describe: ____

22. What are contributing reasons that your baby is not fully breastfed at discharge?
(You may tick more than one box)

- I did not have enough milk 1
- I've stopped because of wounds, cracks or sore nipples 2
- I've stopped because of mastitis 3
- I have inverted/flat nipples 4
- My baby could not/would not breastfeed 5
- My baby was not getting satisfied by breastfeeding 6
- My baby was not gaining enough weight by breastfeeding 7
- I need to know exactly how much my baby gets 8
- I feel bound to breastfeeding 9
- Other 10

Please describe: ____

23. If you have any suggestions or ideas for how breastfeeding could have succeeded for you, please write it here?

____
____
____
____
____
____

Questions about the use of a nipple shield and pacifier for your premature baby

24. Have you used a nipple shield to breastfeed your preterm baby?

Yes 1

No *(proceed to question **30**)* 2

I don't know *(proceed to question **30**)* 2

25. What was the main reason that you used a nipple shield?
(You may tick more than one box)

My baby slipped the nipple 1

My baby became frustrated at the breast 2

My baby fell asleep at the breast 3

My baby could not open his/her mouth high enough to latch on 4

My nipples are inverted/flat 5

My breasts were too engorged 6

My nipples were sore because of wounds/cracks 7

I was recommended to use a nipple shield 7

Other 8
Please explain: ____

26. What was/were any contributing reason/reasons that you used a nipple shield?
(You may tick more than one box)

My baby slipped the nipple 1

My baby became frustrated at the breast 2

My baby fell asleep at the breast 3

My baby could not open his/her mouth high enough to latch on 4

My nipples are inverted/flat 5

My breasts were too engorged 6

My nipples were sore because of wounds/cracks 7

Other 8
Please explain: ____

There were no contributing reasons 7

27. When was the first time you used the nipple shield for your baby?
Date: _____________ (approx

28. Was the nipple shield a help?

No help 1

A little help 2

Some help 3

A great help 4

A very great help 4

29. Is your baby breastfeeding with the nipple shield at discharge?

Yes 1

No 2

30. Did you use a nipple shield when you breastfed your other children?

Yes 1

No 2

I have not breastfed before 2

31. Has your baby used a pacifier during the hospitalization?

Yes 1

No *(proceed to question **36**)* 2

32. When was the first time your baby was given the pacifier?

Date: ___________ (approx.)

33. Did the use of the pacifier change when your infant began to breastfeed more and was less tube-fed?

Yes 1

No *(Proceed to question **32**)* 2

34. In what way was the pacifier used when your baby was ready to be more active in breastfeeding?

We gave the pacifier without restrictions 1

The pacifier was predominantly used when I was not present in the ward 2

The pacifier was predominantly used during diaper changes/care, 3

The pacifier was predominantly used if my baby was crying during blood tests or other painful procedures

The pacifier was predominantly used when my baby was inconsolable 4

The pacifier was predominantly used during tube-feedings 4

The pacifier was removed completely

Other 5

Please describe: ____

35. Does your baby use a pacifier at discharge?

Yes 1

No 2

36. Has your baby been bottle-fed during hospitalisation?

Yes 1

No *(proceed to question **39**)* 2

37. When was your baby bottle-fed for the first time? Date: ___________ (approx.)

38. Is your baby bottle-fed at discharge?

Yes 1

No 2

Questions about skin-to-skin contact

(With skin-to-skin contact we mean that your baby is only dressed in a nappy, maybe a cap and socks, and maybe an open blouse, but in a way that your baby's stomach, chest and legs are in direct contact with your (or another adult's) bare chest.)

39. Have you had your baby skin-to-skin in the period where your baby was cared for in a cot/bed?

Yes	1
No *(proceed to question **41**)*	2

40. How often has your baby been skin-to-skin after incubator care?

A few times	1
A few times a week	2
Once a day	3
Several times a day	4

41. Who had your baby skin-to-skin (this question replies to the whole hospital stay)?
(You may tick more than one box)

Mother	1
Father	2
Your parents	3
Your siblings	4
The baby's siblings	2
Close friends	3
My baby has not been skin-to-skin	4

42. Did you sleep in the hospital since your preterm baby was born?

Yes	1
No *(proceed to question **49**)*	2

43. Did you sleep in the same room as your baby while your baby was in the neonatal ward?

Yes	1
No *(proceed to question **45**)*	2

44. How large a part of your baby's hospital stay did you sleep in the same room as your baby?

Your baby's whole hospital stay	1
At least 50% of your baby's hospital stay	2
At least one night before your baby's discharge to home	2

45. Did you sleep in another room in the neonatal ward than your baby while your baby was in the neonatal ward?

Yes 1

No *(proceed to question **47**)* 2

46. How large a part of your baby's hospital stay did you sleep in another room in the neonatal ward your baby?

Your baby's whole hospital stay 1

At least 50% of your baby's hospital stay 2

At least one night before your baby's discharge to home 2

47. Did you sleep in another area of the hospital than the neonatal ward while your baby was in the neonatal ward?

Yes 1

No *(proceed to question **49**)* 2

48. How large a part of your baby's hospital stay did you sleep in another area of the hospital than the neonatal ward?

Your baby's whole hospital stay 1

At least 50% of your baby's hospital stay 2

At least one night before your baby's discharge to home 2

Questions about breast milk pumping, test-weighing and discharge

49. Are you still pumping/expressing breast milk?

Yes	1
No	2
I have never pumped my milk	2

If no, when did you stop pumping? Date: ________ (approx.)

50. Has your baby been test-weighed during hospitalization?
(With test-weighing we mean weighing the baby just before and just after a breastfeeding session, to calculate the amount of milk the baby has been breastfed)

Yes, my baby has been test-weighed at most breastfeeding sessions	1
Yes, my baby has been test-weighed a few times	2
No, my baby has not been test-weighed *(proceed to question **54**)*	3

51. Was test-weighing helpful for you

Yes, a big help	1
Yes, a lillte help	2
No, it was not helpful	3
I don't know	4

52. How did you experience test-weighing *You may tick more than one box)*

It was stressful	1
It was annoying	2
It was unnatural	3
It was easier to step out of tube-feedings	4
It was nice to know how much my baby breastfed	4
Other	5

Please describe: ____

53. How was it to do without test-weighing?

It was very easy	1
It was easy	2
It was neither easy nor hard	3
It was hard	4
It was very hard	4

54. Have you been home with your baby while he/she still needed tube-feedings? (often called Early discharge program)

Yes	1
No *(proceed to question **58**)*	2

55. When did your baby go home on the Early discharge program (date)? ___________

56. When did your baby end the Early discharge program (date)? __________

57. How was your baby fed at the end of the Early discharge program?

My baby was only breastfed	1
My baby was both breastfed and bottle-fed	2
My baby was only bottle-fed *(go to question **19**)*	3
Other (eg. Cup, lact-aid)<br>Please describe: ______________________________________	4

58. Do you have an appointment with your health visitor after discharge?

Yes	1
No	2

59. Have you received sufficient help, support and encouragement for breastfeeding from the following persons?

a. Your husband/partner	Yes	1	No	2	Don't know	3
b. Your mother/your parents	Yes	1	No	2	Don't know	3
c. Others in your family	Yes	1	No	2	Don't know	3
d. Friends	Yes	1	No	2	Don't know	3
e. Nursing staff	Yes	1	No	2	Don't know	3

Finally, some questions about your baby's hospitalization

60. From which neonatal ward was your infant discharged to home?

    ____

61. Has your baby been on a ventilator

    Yes 1

    No 2

    If yes: for how many days? approx. _______ days

62. Has your baby been treated with nasal CPAP?

    Yes 1

    No 2

    If yes: for how many days? approx. _______ days

63. Has your baby had an ultrasound examination of the head

    Yes 1

    No 2

64. If yes, what did the examination show?

    It was normal 1

    It was not normal 2

Thank you for taking your time to answer the questionnaire.

If you, at your baby's discharge, are breastfeeding fully or partially, are expressing breast milk or given your baby expressed milk, you will receive a new questionnaire at 1, 4, 6, and 12 months after your baby's estimated date of delivery in order to know if you are still breastfeeding or expressing breast milk. The next
If, at discharge, you are not breastfeeding, expressing breast milk or given your baby expressed milk, you will not be contacted again.

Until then, please remember for how long you are breastfeeding without giving anything else than breast and when you breastfeed for the last time. If you, at your baby's discharge, are expressing breast milk or using a nipple shield, please remember for how long you do this.

The survey is done in cooperation with
Knowledge Centre for Breastfeeding Infants with Special Needs
Copenhagen University Hospital, Rigshospitalet
Department of Neonatology
Copenhagen
Denmark
