# Supplementary material for: Improved exclusive breastfeeding rates in preterm infants after a neonatal nurse training program focusing on six breastfeeding-supportive clinical practices
Source: PLoS One. 2021 Feb 3;16(2):e0245273. doi: 10.1371/journal.pone.0245273 (PMC7857627; doi:10.1371/journal.pone.0245273)
Supplement: S1 Table — Sensitivity analyses of primary and secondary outcomes. (PDF) [file pone.0245273.s005.pdf]

Supporting information 5
Sensitivity analyses of primary and secondary outcomes

Table 2b. Sensitivity analyses of Exclusive breastfeeding at discharge and PMA at establishment of excl breastfeeding

	Control group	Intervention group	Pearson Chi-Square
Exclusively breastfeeding mothers at infants discharge to home, n/N %*	223/370 (60.3)	288/424 (67.9)	0.025
Postmenstrual age at establishment of exclusive breastfeeding, mean weeks (95% CI)*	37.55 (37.35 - 37.75)	37.71 (37.53 - 37.89)	0.240

*Postmenstrual age was available for 215/223 mothers in the control group and 276/288 mothers in the intervention group

Table 3b. Sensitivity analyses of maternal self-reported practices in control and intervention groups

	Control group n/N (%)	Intervention group n/N (%)	Pearson Chi-Square
**Practice related to the infant***			
Used a nipple shield	216/342 (63.2)	212/396 (53.5)	0.008
Minimized use of pacifier during breastfeeding establishment	194/349 (55.6)	228/412 (55.3)	0.945
Daily skin-to-skin contact after incubator care	286/342 (83.6)	355/400 (88.8)	0.042
**Practice related to the mother****			
First breastmilk expression before 6 hours of delivery	121/385 (31.4)	191/455 (42.0)	0.002
Rooming-in for the whole NICU stay	175/392 (44.6)	188/481 (39.1)	0.097
Used test-weighing at most breastfeeds	132/373 (35.4)	139/458 (30.3)	0.123

* Calculated from a maximum of 370 and 423 mothers, respectively in the control and intervention group

** Calculated from a maximum of 421 and 494 infants, respectively in the control and intervention group
