# Supplementary material for: Improved exclusive breastfeeding rates in preterm infants after a neonatal nurse training program focusing on six breastfeeding-supportive clinical practices
Source: PLoS One. 2021 Feb 3;16(2):e0245273. doi: 10.1371/journal.pone.0245273 (PMC7857627; doi:10.1371/journal.pone.0245273)
Supplement: Attachment — Submitted filename: PLOS One review.docx [file pone.0245273.s006.docx]

*Improved exclusive breastfeeding rates in preterm infants after a neonatal nurse training program focusing on six breastfeeding-supportive clinical practices.*

I recommend this manuscript be accepted. The study was exceptionally developed and implemented, the write up is replicable, and the analysis is thorough. The authors addressed each variable with statistical analysis and eloquent discussion.

My only suggestion is to define exclusive breastfeeding at discharge. Did this mean the infant was exclusively breastfed the same day as discharge, within 24 hours of discharge, or some other measure?
