# Supplementary material for: Improved exclusive breastfeeding rates in preterm infants after a neonatal nurse training program focusing on six breastfeeding-supportive clinical practices
Source: PLoS One. 2021 Feb 3;16(2):e0245273. doi: 10.1371/journal.pone.0245273 (PMC7857627; doi:10.1371/journal.pone.0245273)
Supplement: Attachment — Submitted filename: Response to reviewers.docx [file pone.0245273.s007.docx]

Response to reviewers

*When we refer to line numbers, please see the version with track changes.*

| **Answer to academic editor** | | |
| --- | --- | --- |
| 1. Please ensure that your manuscript meets PLOS ONE's style requirements, including those for file naming | | We made sure the manuscript meets PLOS ONE’s style requirements, including those for file naming. |
| 2. Please attach a Supplemental file of the training materials used in this study or a link if it's published online. | | We have added the training program (in English) as Supporting information 1. In the supporting information there is a link to the complete Danish material. |
| 4. We note that you have included the phrase “data not shown” in your manuscript | | We have added the sensitivity analyses as Supporting information 5. |
| 3. We note that you have indicated that data from this study are available upon request. PLOS only allows data to be available upon request if there are legal or ethical restrictions on sharing data publicly. For information on unacceptable data access restrictions, please see <http://journals.plos.org/plosone/s/data-availability#loc-unacceptable-data-access-restrictions>.   In your revised cover letter, please address the following prompts:   a) If there are ethical or legal restrictions on sharing a de-identified data set, please explain them in detail (e.g., data contain potentially identifying or sensitive patient information) and who has imposed them (e.g., an ethics committee). Please also provide contact information for a data access committee, ethics committee, or other institutional body to which data requests may be sent.   b) If there are no restrictions, please upload the minimal anonymized data set necessary to replicate your study findings as either Supporting Information files or to a stable, public repository and provide us with the relevant URLs, DOIs, or accession numbers. Please see <http://www.bmj.com/content/340/bmj.c181.long> for guidelines on how to de-identify and prepare clinical data for publication. For a list of acceptable repositories, please see <http://journals.plos.org/plosone/s/data-availability#loc-recommended-repositories>.  We will update your Data Availability statement on your behalf to reflect the information you provide. | | We have uploaded the minimal anonymized data set necessary to replicate our study findings to Figshare. To keep confidentiality, we have given the data as categorical data and in different sheets in the uploaded file, each sheet with random order of participants. All data necessary to replicate results of the NICUs is added in Table 5.  Please update our Data Availability statement on our behalf to reflect the information provided and with new DOI and link. Figshare suggests only to provide the DOI, so please delete the link if you agree.  *Our suggestion to changes:*  *The authors confirm that, the data about improvement of exclusive breastfeeding, postmenstrual age at breastfeeding establishment, changes in six clinical practices, and characteristics of participants are available on Figshare DOI* [10.6084/m9.figshare.13251347](https://doi.org/10.6084/m9.figshare.13251347) <https://figshare.com/articles/dataset/Data_Improvement_of_exclusive_breastfeeding_in_preterm_infants_2/13251347> |
| **Answers to Reviewer #1:**  Thank you for your positive feedback, your questions, comments and concerns that helped us improve the manuscript. We have answered to each point below. | | |
| 1. The study rational, methods, results and discussion focuses only on exclusive BF rates. It could be interesting to bring some considerations on any BF rates, as even partial BF is still beneficial: what were the rates of total (exclusive and partial) BF? Did the authors record them? If so, did they observe any difference after the intervention? | Thank you for your relevant comment on this. We have now added different methods and types of feeding in Table 2 (line 303) and the following text in the results section (line 301):  *“Rates of partial breastfeeding and no breastfeeding decreased, and rates of exclusive breastmilk fed infants increased (Table 2).”*  We think however, that exclusive breastfeeding is the optimal goal also for preterm infants and this study shows that it is achievable. | |
| 2. Also, the main outcome “exclusive BF” was defined in this study as “infant feeding directly at and from the breast” (page 5, line 95), which differs from the WHO definition (“no other food or drink, not even water, except breast milk, including milk expressed or from a wet nurse”, see also <https://www.who.int/nutrition/topics/infantfeeding_recommendation/en/>). I do not understand the choice of this rather restrictive definition. In all cases, it would be important to provide additional information about potential differences in reported rates of exclusive BF when using the WHO definition, which remains a reference in reporting and comparing BF rates. | Thank you for your suggestion. We choose the strongest definition as infants establishing direct breastfeeding continue any breastmilk feeding for a longer duration than infants exclusively breastmilk fed by other methods at the time of discharge (Maastrup 2014b, Keim 2017). We do clearly define exclusive breastfeeding and the study is thereby transparent. We added a reference for the definition (line 101).  Added in Definition of terminology line 103:  *“Partial breastfeeding included other feeding methods in addition to breastfeeding (i.e. bottle, cup, lact-aid) regardless of content. Breastmilk fed included breastfeeding and breastmilk given by other feeding methods.”*  And added in the Background line 59:  *“**Establishing exclusive direct breastfeeding is crucial for preterm infants. It is associated with longer duration of breastmilk feeding as compared to the duration of breastmilk feeding for infants feeding some or all of their exclusive breastmilk diet from a bottle at the time of discharge [14, 15].”*  We consider ‘breastmilk feeding’ (suggested by the reviewer) to be inappropriate as outcome in the present study, when some of the clinical practices aim to affect breastfeeding rather than breastmilk feeding (nipple shield use, pacifier use, test-weighing). The following paragraphs have now been added in the “Limitation section” line 430:  *“Another strength was the narrow definition of breastfeeding as exclusive and direct breastfeeding. Clinical practices such as nipple shield use and pacifier use could have an impact on the infant’s ability to establish exclusive breastfeeding whereas the mother’s milk supply might not be affected. As a result, exclusive breastfeeding was the primary outcome of interest rather than exclusive breastmilk feeding or any breastfeeding.*” | |
| 3. Why the authors choose to assess BF rates at discharge? Information on BF duration or at least on BF continuation after a couple of weeks/months at home would also have been an added value, regarding the dose effects on one hand and the known risk of BF discontinuation at discharge on the other. | The timepoint “Discharge” for the primary outcome exclusive breastfeeding was chosen because this was the most relevant and visible outcome for the neonatal nurses. After discharge many other variables than the practices in the neonatal ward could have stronger effects on breastfeeding duration. This study explored Excl BF when neonatal nurses improved their practice.  No changes were made in the manuscript. | |
| 4. What were the reasons for restricting the training to nurses rather incorporating all the health caregivers, including physicians? There is indeed a global and recognized need to improve medical education and involvement in BF support, a growing interest to conduct interdisciplinary actions, especially for BF issues, and to avoid discordant information to the mothers. Such a training program could have been a synergic opportunity to reach these goals too. | Restricting the training to neonatal nurses (including the few assistant nurses working in the NICUs) was a pragmatic decision. As described in “Settings”, breastfeeding guidance is a core element of neonatal nursing in the participating NICUs and physicians most often consult the nurses about this subject. We know that an interdisciplinary solution would have been stronger, but we assumed that requesting physicians to be included in the training would have decreased the number of NICUs willing to participate in the study.  No changes were made in the manuscript. | |
| 5. Although multicentric, the study is not international, but limited to the Denmark. As in other Scandinavian settings, BF epidemiology and determinant factors in DK are not easy to generalize to the rest of the Europe and other countries. Thus, some information on BF policy in DK, such as duration of the maternity leave or BF rates in the general population, could help in transposition of the results and comparisons with other settings. | Thank you for noticing this. We have now added the following paragraph in “Settings” (line 118):  *“Parents in Denmark have the right to paid parental leave for a total of 52 weeks, 18 weeks solely for the mother and 2 weeks solely for the partner. The reminding weeks can freely be shared between the parents [34]. About 97% of Danish infants initiate breastfeeding [8] and 61% are exclusive breastfed for four months [35].”* | |
| 6. Study design: in order to avoid cross-contamination, the study followed a quasi-experiment design. However, a some more robust alternative in this situation could have been to use a stepped wedge cluster randomised trial (Hemmin, BMJ 2015;350:h391/ doi:https://doi.org/10.1136/bmj.h391). Did the investigators consider this possibility in planning the study, and, if so, why was this strategy not retained? | A stepped wedge cluster randomized design was not feasible in our study as we needed to deal with some participating infants transferred between high intensive care units and medium/low intensive care units. If a stepped wedge design would have been used, many infants, especially the most preterm, would have been excluded during the study time for risk of spillover effect.  No changes were made in the manuscript. | |
| 7. Outcomes: as the study sample was calculated on the basis on the exclusive BF rates increase, this should, on my opinion, be the only primary outcome. Others are secondary outcomes. | Thank you. We have now changed this accordingly | |
| 8. Methods, pages 7-8: The description of the interventional training program for neonatal nurses reports here 5 of the 6 clinical practices, but not the rooming in. Why? Conversely, it comprises the extra attention to give to groups at particular risk of not breastfeeding exclusively, which could also be consider as an additional action on practices. | We understand from your comment that ”co-admission of mothers” is not clearly understood as “rooming-in”, so we added “i.e. earlier rooming-in” to the description of the training (line 161):  *“Work towards more co-admissions of mothers into the NICU i.e. earlier rooming-in”*  We have not described the extra attention to vulnerable groups as a clinical practice, as this would have been hard to measure in the questionnaires answered by mothers. | |
| 9. Although interesting and increasing, early discharge policies with at home care and follow up are not available in most countries. Was the duration of total care (hospital stay and home follow up) recorded and compared between the two periods? This would be interesting from an economical point of view also, as shorter durations would be associated to lower costs, which can make a sound argument for health policies establishments. | Thank you for noticing the missing definition of discharge. We added in the Definitions section (line 106):  “*Discharge was defined as the end of the total period of care from the NICU. If the infant was in an Early Discharge Program, the discharge date was defined as the day the Early Discharge Program ended.”* | |
| 10. More description on participating centres would be useful: except for the four level III, what were the other NICUs levels? How many yearly admissions (min-max)? Among them, were they any Baby Friendly Initiative Hospitals? Availability and use of donor human milk? | We have no information of yearly admissions, but we did report the mean number of beds in the participating NICUs (line 135).  We added level of neonatal care in Table 5.  In description of participants, we added (line 136):  *“all NICUs had access to donor human milk”…*  and  “*No hospitals held a valid BFHI designation as the program was closed in Denmark in 2008.”* | |
| 11. Regarding the rooming-in, parental facilities available accommodations policies in centres would need to be reported too. What kind of accommodations/rooms were offered? Were they similar in all participating centres? | Thank you for this relevant comment. We have now added the following in the description of participants (line 138):  *“Rooming-in was possible in all NICUs, in eight the mother could room-in the infant’s whole NICU stay, in three NICUs at least 50% of the infant’s NICU stay and in two NICUs at least the last night before infant discharge.”* | |
| 12. Was maternal satisfaction assessed and compared between the 2 periods? It would be an important outcome too. | Mothers were asked if they, in their own opinion, received sufficient help, support and encouragement for breastfeeding from the nursing staff, and we agree with the reviewer, that this is important information. However, it was not a predefined outcome for this study, why we have added the information in description of participants (line 289) and not in outcomes of the intervention:  *“Most mothers perceived the help, support, and encouragement from the nursing staff as sufficient (86% in control group and 90% in intervention group).”* | |
| 13. Results, table 5: There were huge differences in nurses participating rates, from one third to 100%. Moreover, 2 centers could not realize the intervention at all. What were the reasons for such differences? What were the putative difficulties in centres with lower adherence? This would help readers to understand and to transpose the feasibility of the intervention. | Thank you for your question. As described in the Participants section (line 133) one NICU did not participate in the intervention, as they could not allocate resources for the training program, and as described in the results section, three NICUs did not implement the breastfeeding meetings, but they participated in the training (line 342).  We don’t know the exact difficulties in these centers, as we did not collect information about the reasons for not adhering to the project.  We added in the Intervention (line 156):  *“The dates were planned in collaboration with the NICU nurse managers, who scheduled the nurses for the training.”*  Number of nurses per NICU have been added in Table 5. | |
| 14. From a methodological and statistical point of view, couldn’t one alternatively consider to include the 2 centres that were unable to perform the intervention in an intention to treat analysis? | Thank you for the relevant comment on this. One NICU did not participate in the intervention and therefor only contributed with mother-infant dyads to the control group.  The three NICUS that did not implement the breastfeeding meetings are included in all the analyses except for breastfeeding meeting attendance ration and duration ratio.  We have now added this in the Statistics (line 241):  *“All infants in the intervention group were included in the analyses irrespective of the wards’ implementation of breastfeeding meetings.”* | |
| 15. Did the investigators perform a separate analysis for each center level? | We have reported breastfeeding rates for each NICU in control and intervention groups (Table 5), but the study was not powered to test significant differences in each ward between control and intervention infants. | |
| 16. A limitation of the study is the relatively high number of lost data, especially in self-reporting questionnaires, encompassing risks of bias, especially reporting bias. Was any action attempted to improve the responding rates (such as phone call by the investigators, others)? | Yes, actions were taken to improve responding rates.  We have now added this in “Data collection” (line 203):  *“Mothers were reminded to answer the questionnaire alternately by text message and e-mail at least three times. To improve participation rate in the intervention group, most of the mothers who did not answer* *the questionnaire within three reminders were called by phone to complete the questionnaire as a telephone interview.* “  and added this in Limitations (line 451):  *“Even though efforts were made to increase response rate, several participants did not complete the study. However, the response rates were similar between control and intervention groups (74% and 73%, respectively).”* | |
| 17. The fact that the use of pacifier, in particular, was not impacted is interesting, as it is thus unlikely to have played a determinant role in this study. Authors could briefly discuss this, considering the existing controversies on the use of pacifiers in NICUs, which could also have beneficial effects in neonatal wards, such as reducing pain and stress, favouring non-nutritive suction, or reducing the risk of sudden infant death. | As described in the Background, former studies have shown associations between the six clinical practices and exclusive BF at discharge. That is why we aimed to improve these six practices.  This manuscript did not assess whether the six practices were associated with exclusive breastfeeding at discharge in the participating infants, and therefore our analyses cannot contribute to this discussion.  We don’t know if the increase in breastfeeding rates would have been more pronounced if more infants had the use of pacifiers minimized during breastfeeding establishment. So, we cannot say that pacifier use is unlikely to play a determinant role in exclusive breastfeeding.  We did, however, mention the controversies in the Background section (line 73):  *Findings from studies of pacifier use are contradictive with some suggesting that pacifier use in preterm infants is associated with earlier transition to exclusive breastfeeding [19] and that minimizing the use of pacifiers during the period of breastfeeding establishment is protective to exclusive breastfeeding [7, 13].*  No changes were made in the manuscript | |
| Minor: 18. Abstract, page 2, lines 26-30: what is primary and secondary outcomes should be made explicit and exposed in this order (see also point 7). | Order changed (line 27 and 89) | |
| 19. Negative results, such as the absence of difference in postmenstrual age at establishment of exclusive BF, should also be mentioned. | We did mention this in the abstract (line 37) and in the result section (line 300). | |
| 20. Introduction, page 4, lines 50-51: Other important protective effects of mother’s milk such as BPD or neurodevelopmental advantage could also be mentioned. | We have added *“and improve neurodevelopment”* (line 53), but the latest systematic review on protective effects of breastmilk on BPD does not show strong evidence.  [Villamor-Martínez](https://pubmed.ncbi.nlm.nih.gov/?sort=date&size=200&term=Villamor-Mart%C3%ADnez+E&cauthor_id=31275904) 2019 Mother's Own Milk and Bronchopulmonary Dysplasia: A Systematic Review and Meta-Analysis  **Conclusion:** To our knowledge, this is the first systematic review and meta-analysis that specifically evaluates the role of MOM on BPD. Our data indicate that MOM may reduce the incidence of BPD when used as an exclusive diet, but this result needs to be interpreted with caution. | |
| 21. Methods, page 9, line 172: Three questionnaires are cited, but only two are described and provided in the supplementary material. | Thank you for noticing. We changed the text to *“Two questionnaires”* (line 191). | |
| 22. The whole intervention could also be considered as a “ bundle of care” which are gaining increasing interest, not only in nosocomial infection control, but also in BF and other practices assessment. This could be mentioned somewhere. | Thank you for this suggestion.  We tested six practices, so in a way we have worked with “bundle of care”. However, we prefer to discuss it as a multicomponent intervention in the present study, since prior studies in the field has used this well-known term (35). | |
| 23. Training and meetings: did study nurses or coordinators supervise them? | We added this to the Training program for neonatal nurses (line 157):  “*The training was provided by the principal investigator in all NICUs* “  We added this to Data collection (line 212):  *“Two or more contact nurses were appointed in each participating NICU. They registered all preterm infants admitted to the NICU during the study periods, informed and included mother-infant dyads in the study, and performed and registered the breastfeeding meetings. Encouraging e-mails were sent to the contact nurses and nurse managers at 2-3 months intervals to improve adherence to the study. The contact nurses could contact the principle investigator with questions and need for support.”* | |
| 24. Results, Table 1: quantitative variable are presented in categorical ranges, which are not always very intuitive. An additional description (even in text) of a few quantitative variables in terms of means and/or median and min-max range and/or DS would be helpful, at least for Gestational age and Birthweight. | When reporting gestational age of participants, we think it is most informative to report it in gestational age groups as the GA will not be normally distributed.  We are not reporting birthweight (associated with GA).  All data reported must be publicly available. By categorizing the data, we are better able to keep anonymity of participants (as Denmark is a small country) and still give a characteristic of participants.  We added range in the results (line 286):  *“GA ranged 24^3^ – 36^6^ in control group and 23^2^ – 36^6^ in intervention group”* | |
| 25. Results, flow chart and figures: not sharp in the pdf version. | Figures have been improved. | |
| 26. Results, Table 2, 3 and 5: not fully visible in the pdf (right columns are cut). | Table 2 is reorganized. We left a comment in the version with tracked changes to have a wider page, and table 3 and 5 should be visible to you in this version. We are following the instructions from the author guideline. | |
| **Answer to reviewer #2.** Thank you for your positive feedback and relevant suggestion. | | |
| Reviewer #2: The study was exceptionally developed and implemented, the write up is replicable, and the analysis is thorough. The authors addressed each variable with statistical analysis and eloquent discussion.  My only suggestion is to define exclusive breastfeeding at discharge. Did this mean the infant was exclusively breastfed the same day as discharge, within 24 hours of discharge, or some other measure? | We have now added the following to the Definition of terminology (line 101 in the version with tracked changes):  “Exclusive breastfeeding was defined as an infant solely feeding directly and from the breast *(Labbok 2012) on the day of discharge.”* | |
